# Modified Surgical Techniques for Managing Intraoperative Floppy Iris Syndrome

**DOI:** 10.1155/2016/1289834

**Published:** 2016-11-23

**Authors:** Pornchai Simaroj, Kaevalin Lekhanont, Puwat Charukamnoetkanok

**Affiliations:** ^1^Department of Ophthalmology, Ramathibodi Hospital, Faculty of Medicine, Mahidol University, Bangkok, Thailand; ^2^Department of Ophthalmology, Mettapracharak (Wat Rai Khing) Hospital, Nakhon Pathom, Thailand

## Abstract

*Purpose.* To report a modified surgical strategy in the management of intraoperative floppy iris syndrome-associated iris prolapse.* Methods.* Prolapsed iris is left as is and a new corneal incision near the original wound but at a different site is created. Depending on the location of the original incision and the surgeon's preference, this additional incision can be used as a new port for phacoemulsification tip or can be the new site for the iris to securely prolapse, allowing for the surgery to proceed safely.* Results.* We present 2 cases of iris prolapse and inadequate pupil dilation in patients with IFIS. Along with our modified technique, additional iris retractors were placed to increase the workspace for the phacoemulsification tip. The cataract surgery was performed successfully without further complications in both cases.* Conclusion.* This surgical technique could be an adjunct to allow the surgeons to expand the armamentarium for the management of IFIS-associated iris prolapse.

## 1. Introduction

Intraoperative floppy iris syndrome (IFIS) was first described in 2005 by Chang and Campbell as a triad of intraoperative signs: (a) billowing of a flaccid iris stroma, (b) a propensity for iris to prolapse toward the phaco- and site-port incisions, and (c) progressive intraoperative pupil constriction [1]. IFIS is classified as severe (when all 3 features are present), moderate (billowing and intraoperative miosis), and mild (iris billowing only) [1]. Systemic alpha-1 antagonists associated IFIS, especially tamsulosin, were reported [2]. Chang and Campbell also postulated that systemic tamsulosin blocked contraction of the smooth muscle of iris dilator that reduced muscle tone causing poor pupil dilation, iris floppiness, and a propensity to prolapse [1]. Several procedures to manage the iris problems in IFIS have been proposed including the use of highly viscous or viscoadaptive ophthalmic viscosurgical devices (OVDs) in conjunction with low-flow surgical techniques, the use of bimanual microincision phacoemulsification, and the placement of iris retractors [3]. Intracameral injection of alpha-agonist drugs (phenylephrine or adrenaline) has also been shown to be effective for preventing IFIS [4, 5]. Although some advocated the use of preoperative topical atropine to maximize cycloplegia in eyes at risk for IFIS, additional intracameral phenylephrine or iris retractors might be required [3]. The purpose of this study is to report a modified surgical strategy in the management of IFIS-associated iris prolapse, besides medications.

## 2. Materials and Methods

**Figure 1:**
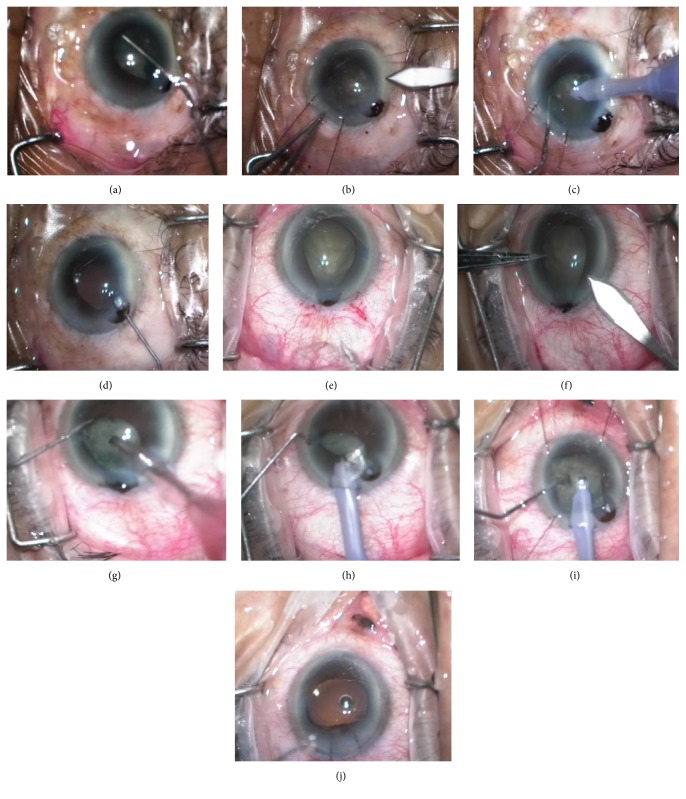
Photographs demonstrating our surgical technique to manage IFIS. In Patient 1, the iris prolapsed during hydrodissection and hydrodelineation (a). Prolapsed iris was left incarcerated in the first incision. Two iris retractors were placed to ensure adequate pupil size and the second main incision was made at 12 o'clock (b). Phacoemulsification was continued via the new superior incision (c). Prolapsed iris tissue was repositioned with OVDS after IOL implantation and OVDS removal (d). In Patient 2, during hydrodissection, the iris prolapsed through the main incision, similar to Patient 1 (e). The second main incision was created lateral to the original incision (f). Phacoemulsification was attempted via the new incision but the pupil started to constrict (g). Phacoemulsification was then performed via the original main wound instead, leaving the iris prolapsed through the second main wound. Nonetheless, the pupil was getting more constricted (h). Three iris retractors were placed and the surgery was continued without further complications (i). Each main wound was sutured at the completion of surgery (j).

Our technique is similar to Tint's. Tint et al. used a single stab incision posterior to phacoemulsification wound, and a single iris retractor was placed [6]. However, if the original incision is too close to the limbus, it will be difficult to do a second stab incision posterior to it. Our technique leaves prolapsed iris as is and creates a new incision near the original wound but at a different site. Depending on the location of the original incision and the surgeon's preference, this additional incision can be used as a new port for phacoemulsification tip or can be the new site for the iris to securely prolapse allowing for the surgery to proceed safely (Figure 1). Additional iris retractors can be used as necessary.

### 2.1. Surgical Techniques

#### 2.1.1. Case 1

A 62-year-old-man was evaluated for cataract surgery. The medical history included hypertension, hyperlipidemia, and benign prostatic hypertrophy. Medications were amlodipine, simvastatin, and tamsulosin. Slit-lamp examination revealed bilateral nuclear sclerotic cataracts, clear corneas, deep anterior chambers, and unremarkable fundoscopy. His best correct visual acuity was 6/24 in both eyes. After giving informed consent, phacoemulsification was performed in the left eye under peribulbar anesthesia. The pupil was moderately dilated. A superotemporal incision and a single side-port incision were done. Following hydrodissection, the iris prolapsed through the main incision and the pupil constricted (Figure 1(a)). Prolapsed iris was left in place. Two iris retractors were placed to ensure adequate pupil size and the second main incision was made at 12 o'clock (Figure 1(b)). Phacoemulsification was done via superior incision (Figure 1(c)). Because of the surgeon preference, Simcoe's needle was used for cortex removal and an intraocular lens (IOL) was implanted under OVDs. Iris retractors and OVDs were removed. Prolapsed iris was repositioned with OVDs and BSS (Figure 1(d)). Stromal hydration was performed gently. Four weeks postoperatively, the examination revealed minimal anterior iris tissue loss but no transillumination defect and the refraction was −0.50–1.0 × 90 with the BCVA of 6/6.

#### 2.1.2. Case 2

A 77-year-old man presented with decreasing vision in the right eye. He had cataract surgery in his left eye 5 years ago. The medical history included hypertension and prostatic carcinoma after radiotherapy for 2 years. Medications included amlodipine and alfuzosin (Xatral XL®). His best corrected visual acuity (BCVA) was 6/24 in the right eye and 6/6 in the left eye. Cornea and anterior chamber depth were within normal parameters. Nuclear sclerotic cataract was found in the right eye and IOL was found in the left eye. Dilated fundoscopy was unremarkable.

After obtaining informed consent, the patient underwent phacoemulsification with peribulbar anesthesia in the right eye by a third-year resident under supervision of the author (PS). The pupil was well dilated preoperatively. A temporal clear cornea incision and a single side-port incision were constructed. Continuous curvilinear capsulorhexis (CCC) was performed under OVDs. During hydrodissection, the iris prolapsed through the main incision (Figure 1(e)). A second main incision was made inferior to the first one (8 o'clock) (Figure 1(f)). Phacoemulsification was tried via the second main wound but the pupil constricted slowly (Figure 1(g)). We decided to switch the surgeon. The prolapsed iris was repositioned with OVDs and phacoemulsification was continued via the original main wound (Figure 1(h)). After insertion of the phacoemulsification tip, the iris prolapsed through the new incision and the pupil constricted further. The phacoemulsification tip was removed and three iris retractors were placed (Figure 1(i)). The surgery was completed without further complications. Prolapsed iris was repositioned with OVDs. Each wound was secured using a single 10-0 nylon suture (Figure 1(j)). The residual OVDs were removed by needle irrigation and aspiration.

## 3. Discussion

IFIS is not an uncommon intraoperative complication and is often associated with the use of adrenergic antagonists such as tamsulosin. The overall prevalence is 1.5–2.5%, and the incidence of IFIS in patients treated with systemic alpha-1 antagonists ranges from 40 to more than 80% [7]. Iris prolapse can occur suddenly following hydrodissection particularly in cases of posterior limbal incision. Trauma to iris can occur during the manipulation to reposition the prolapsed iris causing iris tissue loss, bleeding (hyphema), infection, inflammation, postoperative iris transillumination defects, or monocular diplopia. Furthermore, IFIS may cause reduction of the pupil size that adds difficulty to the surgery.

Generally, certain basic surgical principles such as careful construction of appropriately sized incisions, the use of very gentle hydrodissection, lowering the irrigation inflow rate if possible, and directing irrigation currents away from the pupillary margin should be universally applied to prevent IFIS [3]. Numerous approaches including pharmacologic agents, the use of highly viscous or viscoadaptive OVDs, and placement of mechanical dilating devices have been employed to manage the iris in IFIS [3]. Intracameral adrenaline or phenylephrine usage appeared to be effective in preventing intraoperative miosis or the floppy iris syndrome seen in patients who are on the drug tamsulosin for benign prostatic hypertrophy, during cataract surgery [4, 5]. Positioning iris retractors in a diamond configuration has also been recommended because the retractors maximize exposure immediately in front of the phacoemulsification tip and pull the iris posteriorly away from it [3]. Tint et al. suggested placement of a single stab incision posterior to phacoemulsification wound with one iris retractor under the main incision [6]. However, this technique may be difficult in cases where the original incision is too close to the limbus.

Our technique is primarily aimed at naturally converting the crisis of iris prolapse into instrument-free iris retractor. A new main incision was placed at the most suitable position. Additional iris retractors were used when the pupil was small or constricted during the surgery. With these methods, the surgeon was able to continue and safely finished the operation. At the end of surgery, the trapped iris was gently freed and repositioned with OVDs and the wound was secured with minimal stromal hydration or corneal suture. This technique minimizes the trauma to the iris and reduces the chance of other intraoperative complications. Nonetheless, because of the wide range of IFIS severity, each different technique may be appropriate for each individual. Furthermore, using combination or multiple techniques may be needed if a single method alone was not sufficiently safe. Therefore, this surgical technique could be an adjunct to allow the surgeons to expand the armamentarium for the management of IFIS-associated iris prolapse.

## 4. Conclusions

Creation of an additional clear corneal incision as a new phacoemulsification wound could be a useful surgical strategy in the management of IFIS-associated iris prolapse during phacoemulsification cataract surgery.
